# Case Report of a Patient with Left Ventricular Assistance Device Undergoing Chemotherapy for a New Diagnosis of Lung Cancer

**DOI:** 10.1155/2015/163727

**Published:** 2015-03-22

**Authors:** Maliha Khan, Anum Wasim, Aibek E. Mirrakhimov, Blaithin A. McMahon, Daniel P. Judge, Linda C. Chu, Ashtami Banavali, Amer M. Zeidan

**Affiliations:** ^1^Department of Internal Medicine, Presence Saint Joseph Hospital, Chicago, IL 60657, USA; ^2^Dow University of Health Sciences, Karachi, Sindh 74200, Pakistan; ^3^Johns Hopkins University, Baltimore, MD 21287, USA; ^4^Yale University, New Haven, CT 06510, USA

## Abstract

The optimal management of cancer in patients with severe heart failure is not defined. This issue is particularly challenging when a diagnosis of limited-stage small cell lung cancer (SCLC) is made incidentally in the context of evaluating patient for candidacy for cardiac transplantation. Limited-stage SCLC is typically managed on a curative therapeutic paradigm with combined modality approach involving chemotherapy and radiation. Even with excellent performance status and good organ function, the presence of severe cardiomyopathy poses significant challenges to the delivery of even single modality approach with chemotherapy or radiotherapy, let alone the typical curative combined modality approach. With mechanical left ventricular devices to provide cardiac support, treatment options for cancer in the setting of advanced heart failure may be improved. Here we discuss the therapeutic dilemma involving a patient with severe cardiomyopathy and left ventricular assistant device (LVAD) who was found to have limited-stage SCLC during the evaluation process for cardiac transplantation.

## 1. Introduction

The current era of novel therapeutics has enabled groundbreaking consequences in the world of medicine as survivorship rates from life-threatening conditions are improving. The implantation of left ventricular assistant devices (LVAD) is one such trailblazing treatment modality for heart failure. The LVAD has emerged as a bridge to transplantation until a donor heart is available as well as destination therapy in patients unfit for cardiac transplantation. This has significantly alleviated the mortality risks related to heart failure [1].

Advanced heart failure requires careful attention to fluid balance and treatments with any potential cardiac toxicity may lead to decompensation and death. Unique challenges may further arise in case of a new cancer diagnosis preceding the LVAD implant. We present a case of limited-stage small cell lung cancer (SCLC) of the lung in a patient with an LVAD, undergoing evaluation for cough while under consideration for cardiac transplantation. This paper aims to discuss the optimal management options for SCLC in light of the comorbidities present.

## 2. Case Presentation

**Figure 1:**
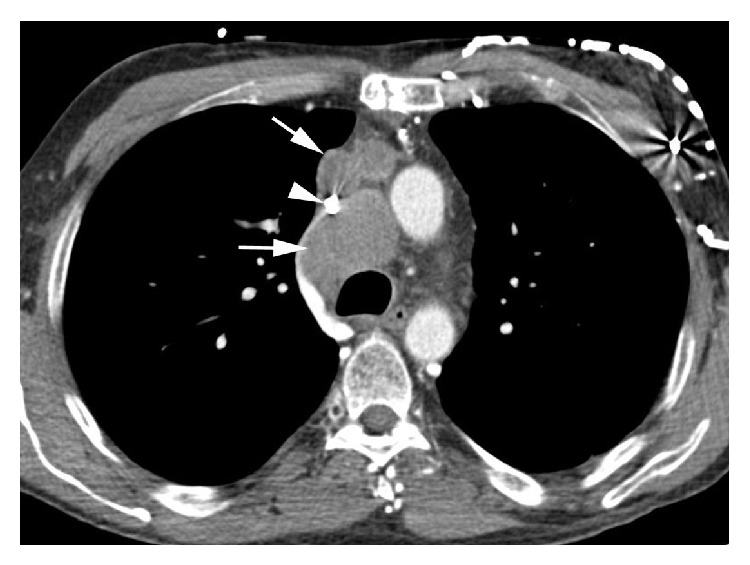
Axial IV contrast-enhanced CT image shows marked right paratracheal and anterior mediastinal lymphadenopathy (arrow) with marked compression of the superior vena cava (arrowhead).

**Figure 2:**
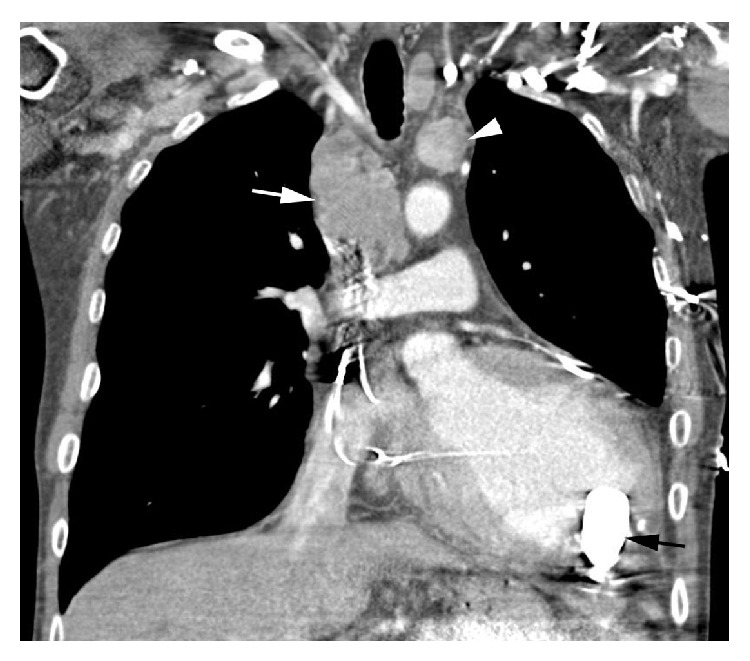
Coronal IV contrast-enhanced CT image shows right (white arrow) and left (white arrowhead) paratracheal lymphadenopathy. LVAD inflow cannula within the left ventricle (black arrow).

We report a 57-year-old male with history of extensive prior tobacco use, nonischemic cardiomyopathy, and an Eastern Cooperative Oncology Group (ECOG) performance status (PS) of 3. After successfully completing his initial evaluation, he was deemed cancer-free and eligible for cardiac transplantation. Due to severe heart failure despite standard medications, he received mechanical cardiac support with an LVAD. Three months after surgery for the LVAD, he was noted to have mediastinal widening on a chest X-ray performed for dyspnea and cough. CT scans showed mediastinal lymphadenopathy without evidence of disease outside the chest (Figures 1 and 2). Renal and liver functions were within normal limits. The patient underwent mediastinoscopy and the pathologic examination was consistent with SCLC. Due to headaches and distended neck veins, he was evaluated for superior vena cava syndrome. The patient was removed from active consideration for cardiac transplantation. After extensive discussion with patient and his family, chemotherapy was administered while hospitalized for close monitoring. He received carboplatin (area under the curve (AUC) 5) on day 1 and intravenous (IV) etoposide 100 mg/m^2^ on days 1–3. Patient received IV dolasetron 100 mg for 30 minutes on days 1–3 and IV prochlorperazine 10 mg every 6 hours as needed. The patient did not receive any prophylactic antibiotics. Carboplatin was used instead of cisplatin due to concerns over aggressive hydration and inducing volume overload. The radiation oncologist had an extensive discussion with the patient and the multidisciplinary team including the LVAD manufacturer and provided the information about the risks, benefits, and complications of concurrent radiation treatments. The patient ultimately decided not to pursue any radiation treatment.

The treatment course was complicated by cellulitis, neutropenic fevers with pseudomonas aeruginosa infection, and protracted nausea and vomiting. The patient was treated with prolonged IV antibiotics course including aztreonam and ciprofloxacin. The patient received oral dolasetron 100 mg on day 4 and subcutaneous pegfilgrastim 6 mg on day 4 after completion of chemotherapy. Anticoagulation with warfarin was started. Although the renal function remained within normal limits, the patient developed signs of worsening overload with left-sided pleural effusion and peripheral edema. Subsequently he became weaker with weight loss of about 25 pounds. After his first cycle of chemotherapy, the patient elected not to receive further chemotherapy and workup. Patient was discharged home under hospice care and passed away about 6 months after the administration of chemotherapy.

## 3. Discussion and Conclusion

To our knowledge, this case represents the first reported circumstance of chemotherapy administration to a patient with LVAD. As expected, chemotherapy administration was complicated by different challenges imposed by the severely compromised cardiac function in a patient with potentially curable cancer. With advanced technology and care, patients with an LVAD can be expected to survive for several years, and therefore this situation might be encountered more often and more data is needed to better understand the best ways to administer chemotherapy in this setting. Development of evidence-based guidelines for use of chemotherapy and radiotherapy use in this situation will likely be difficult due to lack of availability of high-quality data and management will have to rely on expert opinions, personal experience, and individualized patient choices. A multidisciplinary approach of care involving experienced providers (cardiologists, oncologists, radiation oncologists, pulmonologists, and others) in a tertiary specialized center is warranted for optimal outcomes.

SCLC is divided into limited and extensive stage disease. The limited-stage disease is confined to an ipsilateral hemithorax which can safely be encompassed within a tolerable radiation field. The standard chemotherapy regimen consists of etoposide and a platinum agent [2]. Carboplatin is often used in place of cisplatin as it is known to reduce the risk of emesis, neuropathy, and nephropathy. However, the use of carboplatin carries a greater risk of myelosuppression [3]. Carboplatin does not require large fluid administration making it preferable in heart failure patients while cisplatin administration in contrast requires prolonged hydration of large amounts of fluid to maintain renal function [3, 4]. Cisplatin use has also been associated with cardiotoxicity including myocardial infarction, cerebrovascular ischemic events, acute venous thrombotic events, and Raynaud's phenomenon [5]. Combined modality with chemotherapy and thoracic radiation therapy has been known to improve overall median survival of 5% at 3 years in patients with limited-stage SCLC disease [6].

In this particular case, management was complicated by the lack of relevant medical literature regarding optimal oncologic therapy for potentially curable limited-stage SCLC in a patient with coexisting LVAD. While isolated cases of SCLC can be successfully managed in the context of the predefined guidelines, planning out a reasonable management approach is, on the contrary, highly deterred in the situation of a coexisting LVAD implant and the complications that can arise as a result.

Malignant and nonmalignant lesions detected in routine imaging in patients with LVAD have been reported and numerous noncardiac surgical procedures have been performed in these patients to date [7, 8]. A case report published in 2011 described a 58-year-old female who was implanted with an LVAD despite a prior existing pulmonary nodule, which was later diagnosed as an adenocarcinoma [7]. A lower lung lobectomy was cautiously performed under strict hemodynamic control owing to the challenges posed by the LVAD, stressing upon the dire need for stringent cancer screening and patient selection before LVAD implantation [7]. Wei et al. also recounted a similar case [8]. These patients however were not reported to have received chemotherapy.

Patients with advanced heart failure who are considered for cardiac transplantation are meticulously screened for neoplasms including rectal examination and stool occult blood examination, pelvic examination, and pap smear and mammography for women [9]. Identification of a malignancy not only prevents eligibility for cardiac transplantation but also poses grave challenges regarding treatment for the malignancy including morbid but not fatal infections [10]. Most malignancies with metastatic potential except primary CNS tumors are considered a contraindication to cardiac transplantation, unless successfully treated without recurrence for five years [11]. One such patient underwent a radical prostatectomy to reacquire his transplantation candidacy status [12].

More data should be reported to allow the development of management guidelines for administering chemotherapeutic agents to LVAD patient with concurrent malignancy to allow delivery of best care in the context of a balanced risk benefit index.
